# Warm-up optimization in amateur male soccer players: A comparison of small-sided games and traditional warm-up routines on physical fitness qualities

**DOI:** 10.5114/biolsport.2023.114286

**Published:** 2022-05-10

**Authors:** Rohit K. Thapa, Filipe M. Clemente, Jason Moran, Felipe Garcia-Pinillos, Aaron T. Scanlan, Rodrigo Ramirez-Campillo

**Affiliations:** 1 Department of Sports Biomechanics, Lakshmibai National Institute of Physical Education, Gwalior, India; 2 Escola Superior Desporto e Lazer, Instituto Politécnico de Viana do Castelo, Rua Escola Industrial e Comercial de Nun’Álvares, 4900-347 Viana do Castelo, Portugal; 3 Instituto de Telecomunicações, Delegação da Covilhã, Lisboa 1049-001, Portugal; 4 School of Sport, Rehabilitation and Exercise Sciences, University of Essex, Colchester, Essex, United Kingdom; 5 Department of Physical Education and Sports, University of Granada (Granada, Spain); 6 Department of Physical Education, Sports and Recreation, Universidad de La Frontera, Temuco, Chile; 7 Human Exercise and Training Laboratory, School of Health, Medical and Applied Sciences, Central Queensland University, Rockhampton, Queensland, Australia; 8 Department of Physical Activity Sciences. Universidad de Los Lagos. Santiago. Chile; 9 Exercise and Rehabilitation Sciences Laboratory. School of Physical Therapy. Faculty of Rehabilitation Sciences. Universidad Andres Bello. Santiago, Chile

**Keywords:** Football, Sports, Athletic performance, Exercise, Physical activity, Running, High-intensity interval training, Physical education and training, Warm-up exercise

## Abstract

The aim of this study was to compare the effects of small-sided soccer games (SSSGs) and traditional warm-up (TWU) routines on physical fitness qualities in soccer players. Following a between-subject, randomized design, amateur-level soccer players were assigned to a SSSG warm-up (n = 10; age: 19.3 ± 2.8 years) or TWU group (n = 10; age: 19.3 ± 2.4 years). Players completed multiple trials of 10-m and 30-m linear sprints, change-of-direction speed (CODS) tests, and countermovement jumps (CMJ) prior to and following the warm-up routine. Separate mixed ANOVAs were performed to assess group effects (SSSG vs. TWU), time effects within each group (pre- vs. post-warm-up), and their interaction for each physical fitness quality. No significant interaction effects were observed for any dependent variable. Significant improvements were evident between baseline and follow-up measurements for 10-m sprint time (p = 0.002, Hedges’ g effect size [g] = 0.59) and CMJ variables (height: p = 0.016, g = 0.20; power: p = 0.003, g = 0.19; force: p = 0.002, g = 0.14) in the TWU group and for CODS performance time (p = 0.012, g = 0.51) and CMJ variables (height: p < 0.001, g = 0.46; power: p = 0.002, g = 0.35; force: p = 0.001, g = 0.27) in the SSSG warm-up group. Both SSSG and TWU protocols improved selected physical fitness qualities with SSSG more effective at improving CODS and CMJ performance, and TWU more effective at improving linear speed. Soccer coaches may choose between SSSG or traditional warm-up activities according to player needs and preferences; however, the superior effects of SSSG suggest it might offer greater benefits than TWU in preparing players for optimal physical output.

## INTRODUCTION

A warm-up helps to attain optimal performance [1] and reduces injury risk through various mechanisms such as increasing muscle temperature, nerve conductivity, metabolic reactions, blood flow, oxygen consumption, anaerobic energy provision, and force expression [2, 3] and inducing post-activation performance enhancement [4]. In addition, a warm-up may also provide an opportunity to mentally prepare players for upcoming tasks and induce positive psychological effects such as enhancing focus and self-confidence [1–3].

In soccer, warm-up protocols such as the FIFA 11+ prevention programme [5] or the Sportsmetrics Warm-up for Injury Prevention and Performance [6] commonly include running, static and dynamic stretching [7], neuromuscular activities for preventing injury [8], high-intensity and post-activation performance exercises [4], and drills with specific tactical objectives (e.g., pressing drills if a session’s objective is defending) [9]. In this way, such warm-up protocols enhance performance in transitioning from less specific to more specific tasks, relative to the demands imposed during competitive play. Given the frequent execution of high-intensity actions (e.g., sprinting, change in direction speed [CODS], jumping) in soccer [10, 11], specific warm-up activities may be key to optimizing the physical fitness qualities and skills of players most important for success [12]. In this sense, small-sided soccer games (SSSGs) used as a warm-up activity may optimize the specificity of player preparation as they closely resemble the demands of competition [13].

Though SSSGs have been extensively studied as a training method in soccer [13–16], evidence regarding their acute effects as a warm-up technique is scarce and heterogeneous in male soccer players, with benefits observed to physical fitness qualities (i.e., jump variables, CODS, repeated sprint performance) in amateur players over traditional warm-up (TWU) protocols (i.e. Premier League soccer club-based warm-up routine) reported in one study [9] but not shown in semi-professional players in another study [17]. Moreover, TWU (˜23 min) are typically longer than SSSG when used in a warm-up protocol (˜12 min) [9, 17], which indicates SSSG may offer useful practical benefits for administration of the warm-up phase in a time-efficient manner. In addition, existing research has only examined the effects of SSSG as a warm-up technique on physical fitness qualities in soccer players using 3 vs. 3 team configurations [9, 17] and without consideration of tactical constraints. In turn, SSSGs aimed at specific tactical elements with a greater number of players per team may induce a soccer-specific warm-up stimulation that better represents match-play and physically prepares players for upcoming demands [18, 19] than SSSGs tested to date.

Although some findings [9] on the effects of SSSGs used as a warm-up approach on physical fitness qualities in soccer are encouraging, due to the scarcity of literature and limited methodologies tested (i.e. 3 vs. 3 team configurations), we sought to compare the effects of SSSGs involving a greater number of players and targeting ball possession as a tactical consideration against a TWU. The focus on ball possession as a tactical element was included in our study given it has been popularized in the literature [9, 18, 19], and SSSGs aimed at this specific tactical element with a greater number of players per team, such as 4 vs. 4 with 2 additional players on one team (i.e., 4 vs. 4 + 2) may induce a soccer-specific warm-up stimulation that better represents match-play. From a practical perspective, a 4 vs. 4 + 2 SSSG configuration allows more players in the squad to warm-up together simultaneously than SSSG with less players involved. Further, recent developments in warm-up strategies in soccer suggests that a short-duration (i.e., ˜12 minutes) specific warm-up is as effective as a longer (i.e., ˜20 minutes) warm-up in improving readiness to play a match [20] and physical fitness qualities (i.e., sprint and repeated sprint ability), and may induce lower perceived exertion [21, 22]. Accordingly, to increase the ecological validity of our study, a relatively short warm-up strategy was adopted (i.e., 12 min) for both warm-up protocols. Therefore, the aim of the study was to compare the effects of SSSGs and TWU routines on physical fitness qualities in soccer players. We hypothesized SSSGs as a warm-up approach would induce greater improvements in physical fitness qualities than a TWU in soccer players.

## MATERIALS AND METHODS

### Subjects

Based on a previously reported effect size for CODS (ES = 0.8) following a SSSG warm-up by Zois, Bishop [9] an *a* *priori* analysis using G*Power software (v.3.1.9.4, University of Kiel, Kiel, Germany) indicated that sample of 16 players would be required to obtain an f = 0.4 (large effect), and power = 0.80, with two groups and two measurements. Consequently, twenty-four male players volunteered to take part in the study. The inclusion criteria for the study involved: attendance at the familiarization session and the absence of any major injury in the lower-limbs in the previous 6 months or any other recent injury. Three players did not complete the familiarization session and one player was injured during training prior to data collection. Accordingly, 20 players (age: 19.3 ± 2.6 years; height: 162.3 ± 6.0 cm; body mass: 60.6 ± 8.2 kg) from an amateur India 2nd division league soccer club completed the study (Table 1). An online randomization tool (www.randomizer.org) was used to allocate players to the SSSG (n = 10) and TWU (n = 10) (Table 1). The players were informed about the study details and risks of participation before providing written informed consent. The study was approved by the Departmental Research Committee of Lakshmibai National Institute of Physical Education and the protocol conformed to the Declaration of Helsinki (1964, updated in 2013).

**TABLE 1:** Demographic and aerobic capacity variables between the traditional warm-up (TWU) and small-sided soccer game (SSSG) warm-up groups.

Variables	Overall (n = 20)	TWU Group (n = 10)	SSSG Group (n-10)	p-value
	Mean ± SD			
**Age (years)**	19.3 ± 2.6	19.3 ± 2.4	19.3 ± 2.8	1.00
**Height (cm)**	162.3 ± 6.0	161.5 ± 4.6	163.1 ± 7.4	0.59
**Body mass (kg)**	60.6 ± 8.2	62.1 ± 8.5	59.1 ± 8.1	0.79
**Yo-Yo IR1 distance (m)**	908 ± 246	976 ± 202	840 ± 277	0.23
**VO_2_max_predicted_ (ml/kg/min)**	44.0 ± 2.1	44.6 ± 1.6	43.5 ± 2.4	0.23

*Note:* p-values derived from separate independent t-tests showing groups to be similar across variables; abbreviations ordered alphabetically; SD: standard deviation; VO_2_max_predicted_: predicted VO_2_max; Yo-Yo IR1: Yo-Yo Intermittent Recovery Level 1.

### Procedure

**FIG. 1:**
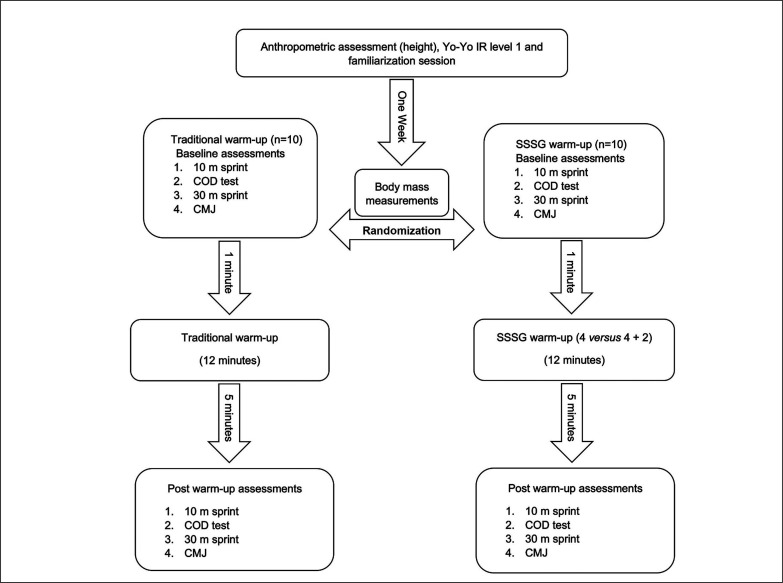
Schematic of the study design. Note: Yo-Yo IR level 1: Yo-Yo Intermittent Recovery Test level 1; SSSG: small-sided soccer game; COD: change-of-direction; CMJ: countermovement jump.

One familiarization session was conducted a week prior to data collection. Standing height, age, and Yo-Yo intermittent recovery level 1 tests scores were recorded on the same day as familiarization. The temperature, humidity, and wind velocity upon commencing the testing session were 19˚ C, 84%, and 4 km/h respectively. Players were asked to refrain from any strenuous activity and alcohol consumption for 24 h and eating for 3 h prior to testing. A between-subject, randomized design was used to compare the effects of SSSGs with a TWU on 10-m and 30-m linear sprint, CODS, and countermovement jump (CMJ) performance. The study was conducted during the start of the pre-season. Players were provided with a day of rest prior to data collection, and assessed on a single day between 9:00 and 11:00. Upon arrival for testing, players underwent body mass measurement, followed by very light jogging (˜60% of self-estimated maximum heart rate intensity) for 5 min, baseline testing, the warm-up protocol (i.e., TWU or SSSG) for 12 min, and follow-up testing after 5 min of passive standing (i.e., to facilitate the resynthesis of high-energy phosphate pools [23, 24]) (Figure 1). The players wore the same football shoes in all the assessments, carried out on natural soccer turf, led by the same assessor.

### Warm-up protocols

### Traditional warm-up

Players completed a series of dynamic stretching exercises across 15 m before jogging back at a self-selected speed to the starting line. In this fashion, players completed two sets of adductor (i.e., internal rotation), abductor (i.e., external rotation), hamstring (i.e., single stiff leg forward bending stretch), gluteal (i.e., hip flexion and extension), and ankle-oriented (i.e., ankle rotation) dynamic stretching exercises. Thereafter, players completed five 15-m sprints at 60% of maximal self-estimated effort (i.e., 100% would represent the perceived maximal speed in a 15-m sprint), three sprints at 80% of maximal self-estimated effort, two sprints at 90% of maximal self-estimated effort, and a final sprint at maximal effort. After each sprint, players jogged back at a self-selected speed to the starting line.

### Small-sided soccer game warm-up

**FIG. 2:**
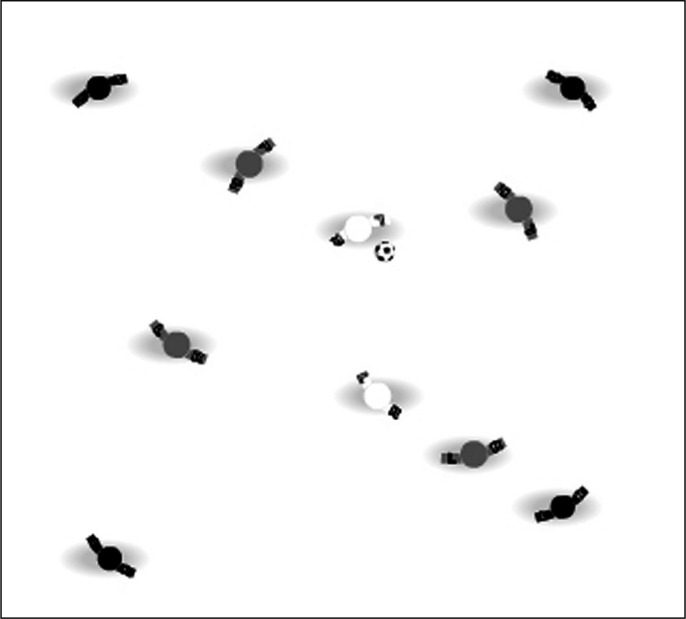
The setup of the small-sided soccer games following a 4 (black) vs. 4 (grey) + 2 (white) neutral players configuration on a 15-m × 15-m pitch. The white players supported the team in possession of the ball.

The SSSG warm-up protocol consisted of a 12-min match on a 15-m × 15-m pitch. The SSSG focused on ball possession with a 4 vs. 4 plus 2 neutral players configuration adopted given its common use in soccer [25, 26] (Figure 2). In addition, SSSGs aimed at ball possession as a tactical element and with a greater number of players per team (i.e., 4 vs. 4 + 2) may induce a soccer-specific warm-up stimulation that better represents match-play and physically prepares players for upcoming demands than configurations (i.e., 3 vs. 3) examined previously [9, 17].

Players were randomly distributed to teams and were instructed to keep possession of the ball for as long as possible. Two licensed soccer coaches supervised the SSSGs. The team in possession of the ball was supported by two neutral players (i.e., replaced after each set, with all players acting as neutral players at some point) to keep possession of the ball, meaning play comprised of 6 vs. 4 players to create a numerical advantage for one team. Six sets of exercise were performed in the SSSG warm-up, with each set consisting of 90 s of active play followed by 30 s of active recovery. During the active recovery, players completed the same dynamic stretches as completed in the TWU group. One of the coaches provided verbal cues (e.g., ‘keep moving’, ‘don’t lose the ball’, ‘make the defenders move’) to the team in possession of the ball. A second coach provided verbal cues (e.g., ‘press’, ‘win the ball’, ‘don’t let them play’) to the defending team. The two coaches timed each SSSG set and active recovery, and ensured a replacement ball was always available.

### Physical fitness test battery

### Linear sprint tests

A 10-m (ICC = 0.79) and a 30-m (ICC = 0.96) linear sprint test was conducted 5 min and 15 min, following completion of the warm-up protocol respectively. A timing system consisting of two pairs of single-beam photocells (Cronox, Madrid, Spain) was positioned at a height of 0.6 m above ground level and players started 0.5 m behind the first photocell with their preferred foot forward on a marked line. Two trials were conducted for each sprint test, with a 1 min passive standing rest between trials. The fastest trial was selected for analysis in each sprint test.

### Change-of-direction speed test

**FIG. 3:**
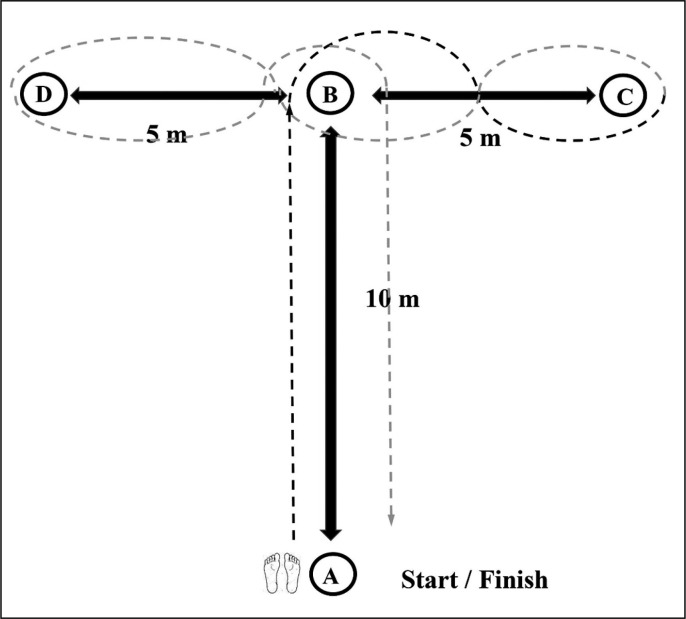
The configuration of the modified version of the agility t-test was used to assess change-of-direction speed. Cones at a height of 0.3 m were placed at points A, B, C, and D with players being instructed to sprint from A to B, perform a 90˚ turn from cone B towards cone C, then perform a 180˚ turn at cone C towards cone B, before performing a turn at cone B towards cone D, then performing a 180˚ turn at cone D towards cone B, and finally returning to the start/finish line. The players were required to weave around the cones without touching them. Trials were discarded if the cones were displaced while changing direction with players being required to repeat the trial after 1 min of passive standing rest. Note: black line denotes primary turns, and grey line denotes secondary turns.

A CODS test was conducted 8 min following completion of the warm-up protocol using a modified version of the the agility T-test (ICC = 0.91) [27], to replicate soccer-specific CODS demands (Figure 3). A timing system with one pair of single-beam photocells (Cronox, Madrid, Spain) was placed at a height 0.6 m above ground level at the start/finish line. Players started 0.5 m behind the start/ finish line in a standing position with their preferred foot forward. Two trials were conducted with 1 min of passive standing rest between trials and the fastest trial selected for analysis.

### Countermovement jump

A CMJ test with the hands on the hip was conducted 20 min following completion of the warm-up protocol. Players were instructed to jump maximally following a countermovement with a self-selected magnitude of knee flexion. Height (CMJ_height_), force (CMJ_force_) and power (CMJ_power_) (all ICC = 0.96) generated during the CMJ were measured with a validated (vs. force plate, jump height: r = 0.99; jump power: r = 0.85) [28] IOS application, My Jump 2, installed on an Apple iPad 8^th^ generation (Apple Inc., California, USA) with a 120-Hz high-speed camera at a quality of 720 p. The camera was directed as low as possible facing each player in the frontal plane ˜2 m away to best record jump performance. Two trials were performed by each player with a recovery period of 1 minute between trials. The highest value for each variable was selected for analysis.

### Statistical analysis

Analyses were conducted using IBM SPSS version 20.0.0 (IBM, New York, USA). The normality of all data was verified using the Shapiro-Wilk test. Data are presented as mean ± standard deviation (SD). A two (baseline vs. follow-up testing) × two (TWU vs. SSSG warm-up) mixed ANOVA was used to analyze the collected data. In addition, paired (within-group comparisons) and independent (between-group comparisons) t-tests were used for post-hoc analyses with Bonferroni adjustments applied. The percentage change score for each variable in each group was calculated using the equation: [(meanpost – mean_pre_)/mean_pre_] × 100. Effects sizes were calculated as partial eta squared (ɳ_p_^2^) for interaction and main effects in each ANOVA and as Hedge’s *g* to assess changes between baseline and follow-up testing in each group. The magnitude of effects for _ɳ_2(p) was interpreted as small (< 0.06), medium (≥ 0.06–0.13), and large (≥ 0.14) [29], while Hedge’s *g* was interpreted as trivial (< 0.2), small (0.2–0.6), moderate (> 0.6–1.2), large (> 1.2–2.0), very large (> 2.0–4.0) and extremely large (> 4.0) [30]. Statistical significance was set at *p* ≤ 0.05.

## RESULTS

**FIG. 4:**
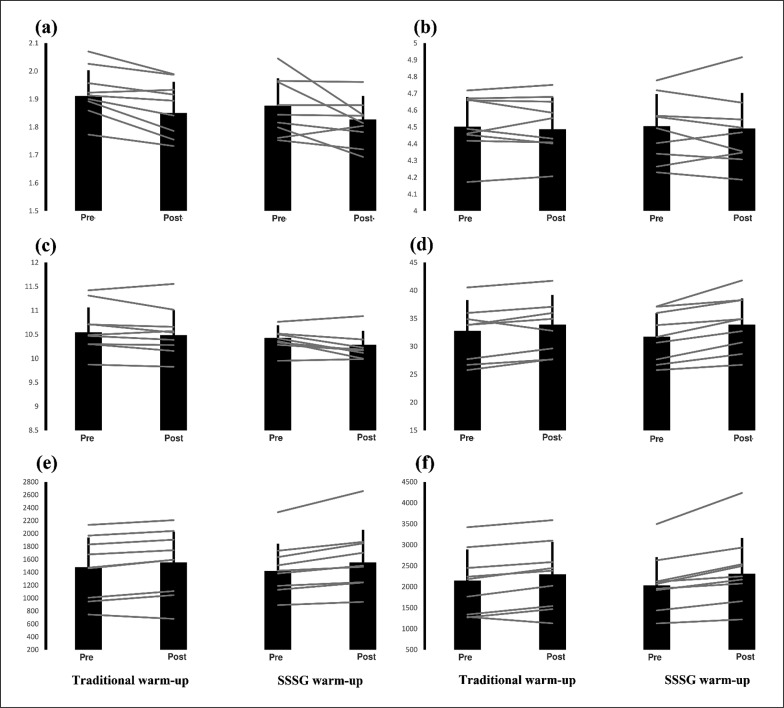
Mean (column) ± standard deviation (error bar) along with individual responses (grey lines) for (a) 10-m linear sprint time [s], (b) 30-m linear sprint time [s], (c) change-of-direction speed test time [s], (d) countermovement jump height [cm] (e) countermovement jump force [N] and (f) countermovement jump power [W] prior to and following a traditional warm-up and small-sided soccer game (SSSG) warm-up protocol.

The mean ± SD for each dependent variable is shown in Table 2, with individual data presented in Figure 4. Statistical outcomes for comparisons in each dependent variable are also shown in Table 2. No significant baseline (*p* = 0.41–0.86) differences were observed between the TWU group and the SSSG group (i.e., independent t-test) in any of the dependent variables further supporting the matching of groups regarding physical fitness qualities.

**TABLE 2:** Statistical comparisons for changes in physical fitness qualities between traditional warm-up and small-sided soccer games (SSSG) warm-up protocols in soccer players.

Variable	Traditional warm-up group (n = 10)				SSSG warm-up group (n = 10)				Main time effect	Time × group
	Baseline	Follow-up	%Δ	*p*-value [*g*] *Magnitude*	Baseline	Follow-up	%Δ	*p*-value [*g*] *Magnitude*	*p*-value [η_p_^2^] *Magnitude*	*p*-value [η_p_^2^] *Magnitude*
	Mean ± SD				Mean ± SD					
**10-m sprint time (s)**	1.91 ± 0.09	1.85 ± 0.11	-3.2%	0.002* [0.59] *Moderate*	1.88 ± 0.10	1.83 ± 0.09	-2.6%	0.076 [0.51] *Moderate*	**0.001^#^** [0.460] *Large*	0.661 [0.011] *Small*

**30-m sprint time (s)**	4.50 ± 0.18	4.49 ± 0.19	-0.3%	0.466 [0.07] *Trivial*	4.51 ± 0.19	4.49 ± 0.21	-0.3%	0.644 [0.06] *Trivial*	0415 [0.037] *Small*	0.951 [<0.001] *Small*

**CODS time (s)**	10.55 ± 0.52	10.49 ± 0.52	-0.6%	0.157 [0.11] *Trivial*	10.43 ± 0.26	10.28 ± 0.29	-1.4%	0.012* [0.51] *Moderate*	**0.003^#^** [0.392] *Large*	0.171 [0.101] *Medium*

**CMJ_height_ (cm)**	32.8 ± 5.5	33.9 ± 5.30	3.4%	0.016* [0.20] *Small*	31.7 ± 4.2	33.9 ± 4.7	6.8%	< 0.001* [0.46] *Small*	**<0.001^#^** [0.671] *Large*	0.078 [0.162] *Large*

**CMJ_power_ (W)**	2149 ± 737	2301 ± 769	7.1%	0.003* [0.19] *Trivial*	2030 ± 674	2310 ± 850	13.8%	0.002* [0.35] *Small*	**<0.001^#^** [0.654] *Large*	0.099 [0.144] *Large*

**CMJ_force_ (N)**	1479 ± 458	1552 ± 478	4.9%	0.002* [0.14] *Trivial*	1419 ± 421	1551 ± 504	9.3%	0.001* [0.27] *Small*	**<0.001^#^** [0.676] *Large*	0.098 [0.145] *Large*

Note: abbreviations ordered alphabetically; CODS: change-of-direction speed; CMJ: countermovement jump; g: Hedges’ *g* effect size; η_p_^2^: partial eta squared; SD: standard deviation; %Δ: percentage change score between baseline and follow-up measures;

* : significant difference between baseline and follow-up measures;

# : significant main time effect.

Separate 2 × 2 mixed ANOVAs revealed no significant group effects (*p* = 0.29–0.99) or interaction effects (time × group) (*p* = 0.08–0.95) for any dependent variable. However, significant time effects were observed in 10-m sprint time (*p* = 0.001, ɳ_p_^2^ = 0.46), CODS test time (*p* = 0.003, ɳ_p_^2^ = 0.39) and CMJ variables (CMJ_height_, *p* = < 0.001, ɳ_p_^2^ = 0.67; CMJ_power_, *p* = < 0.001, ɳ_p_^2^ = 0.65; CMJforce, *p* = < 0.001, ɳ_p_^2^ = 0.68). Post-hoc paired sample t-tests revealed significant improvements in 10-m sprint time (*p* = 0.002, *g* = 0.59) and CMJ variables (CMJ_height_, *p* = 0.016, *g* = 0.20; CMJ_power_, *p* = 0.003, *g* = 0.19; CMJforce, *p* = 0.002, *g* = 0.14) in the TWU group. On the other hand, significant improvements in CODS test time (*p* = 0.012, *g* = 0.51) and CMJ variables (CMJ_height_, *p* = < 0.001, *g* = 0.46; CMJ_power_, *p* = 0.002, *g* = 0.35; CMJforce, *p* = 0.001, *g* = 0.27) were observed in the SSSG warm-up group (Table 2).

## DISCUSSION

The aim of this study was to compare the effects of TWU and SSSG as warm-up approaches on physical fitness qualities in soccer players. Results revealed both warm-up protocols induced improvements in physical fitness qualities deemed important in soccer. However, moderate improvement in 10-m linear speed (3.2%) and small to trivial improvement in CMJ performance (3.4–7.1%) was observed following TWU, while moderate improvement in CODS (1.4%) and small improvement in CMJ performance (6.8–13.8%) was observed following SSSGs. Therefore, the present findings confirm SSSG can be used as a warm-up strategy to acutely improve important physical fitness qualities in soccer players.

Our findings suggest warm-up strategies, in the form of a TWU or SSSG, improve various physical fitness qualities in soccer players. In agreement with our results, previous research has shown various warm-up strategies, such as TWU, 3 vs. 3 SSSG, long specific, short specific, and 5-repetition maximum leg press to improve physical fitness qualities, including linear sprint, vertical jump, reactive agility, and repeated sprint ability in various samples of soccer players [9, 17, 21, 22]. Both warm-up protocols examined in this study likely produced positive acute effects on physical fitness qualities due to various physiological mechanisms, but namely a rise in body temperature which subsequently enhances muscle metabolism [31] as well as increases muscle fiber contractile performance [32] and conduction velocity [33]. In this regard, a 1°C increase in muscle temperature enhances power output and can improve exercise performance such as sprinting, jumping, and maximal strength during knee extension between 2–5% [34–36]. Relatedly, warm-up protocols intended to increase muscle temperature have also been shown to be positively related to movement velocity during 20-s maximal efforts on isokinetic cycle ergometers [35] and relative work rate during cycling [37]. Furthermore, an elevation in oxygen uptake kinetics [38] following dynamic warm-up protocols may enhance the functioning of the cardiovascular system (e.g., increased systolic volume). Consequently, the multidimensional acute benefits of different warm-up protocols likely underpin the benefits we observed for various physical fitness qualities in soccer players.

While both warm-up protocols improved physical fitness, we noted a moderate improvement in 10-m sprint time only in the TWU group, probably attributed to the specificity of the TWU protocol to linear sprinting. Indeed eleven 15-m linear sprints at different intensities were performed in the TWU group, which may have promoted neuromuscular activation and coordination of muscles recruited specifically across 10-m sprints [39]. In support of this notion, literature suggests warm-up activities should be chosen on the degree of their specificity relative to the exercise they precede [3, 39]. In turn, van den Tillaar, Lerberg [22] suggested the mere execution of an extended generalized warm-up did not constitute a sufficient stimulus to improve maximal linear sprint performance, but instead recommended a targeted approach of specific activities that resemble the nature of subsequent performance. Accordingly, the significant improvement in 10-m linear sprint speed in the TWU group but not in the SSSG warm-up group could be explained by the absence of specific linear sprinting activities being employed in the ad hoc match-play involved in the SSSG. In a similar vein, the lack of any improvement in 30-m sprint speed in both the TWU and SSSG groups could be due to the insufficient specificity of the stimulus with neither performing sprints of this distance in the respective warm-up protocols. Similar to our findings, Zois, Bishop [9] reported no significant improvement in 20-m linear sprint performance after a SSSG warm-up following a 3 vs. 3 configuration. Therefore, from a practical perspective, the collective evidence suggests soccer-specific linear sprints (e.g., 10–30 m) should accompany SSSG when used as a warm-up to gain maximal benefits, such as during the active intra-set recovery period interspersed throughout SSSG.

In contrast to 10-m linear sprint performance, only the SSSG warm-up group experienced significant positive improvements in CODS. In line with the principle of specificity discussed for our findings regarding discrepancies in the effects of each warm-up on 10-m linear sprint speed, players executed several changes-of-direction with or without the ball during the SSSG warm-up but not during the TWU. Activity patterns during the SSSG may have activated muscles required for effectively changing direction (e.g., adductor magnus, hamstrings, gluteus maximus, quadriceps) [40] and, in the process, enhanced CODS. Indeed, a meta-analysis indicated that SSSG training improve CODS in team sports athletes such as soccer, handball, rugby, volleyball, and Australian football players [41]. Furthermore, a small pitch area, such as the 15-m × 15-m pitch we utilized, has been suggested to increase the pressure placed on players on offence given there is less space to evade defenders, necessitating fast multi-directional movements to maintain possession [15]. Indeed, Zois, Bishop [9] observed a similar acute improvement (3.8%) in reactive agility speed following a SSSG warm-up, which exceeded the benefit (0.9%) of a TWU consisting of general activities such as butt-kicks, high-knees, and body-weight squats, specific movements such as lateral skipping, back and forth sprinting, and CODS movements, and ball control activities such as dribbling, passing, and run-through. Therefore, the collective evidence suggests SSSG warm-ups are superior to TWU in promoting benefits for CODS.

Our study also demonstrated improvements in CMJ performance with both warm-up protocols. Although no group by time interactions were noted for CMJ variables, the SSSG warm-up induced a two-fold increase in CMJ performance (6.8% CMJ_height_, 13.8% CMJ_power_, 9.3% CMJforce) compared to the TWU (3.4% CMJ_height_, 7.1% CMJ_power_, 4.9% CMJforce). Our results confirm those reported by Zois, Bishop [9] who observed a 3 vs. 3 SSSG warm-up increased CMJ_height_ (6%) more than a traditional team-sport warm-up (< 1%) among amateur male soccer players. Similar findings were also observed among handball players, where a small-sided games based warm-up routine elicited better performance in CMJ than the TWU [42]. The greater improvement in CMJ performance with SSSG warm-up protocols compared to TWU protocols could be attributed to an intensity-dependent relationship [43] whereby warm-up protocols performed at higher intensities have been shown to elicit greater positive effects on jumping performance (i.e., drop jump, CMJ, and squat jump) among volleyball players [43] and basketball players [44]. In this sense, the SSSG warm-up likely required players to perform more frequent high-intensity efforts, including jumps, while undertaking different unpredictable match scenarios compared to the planned TWU protocol. Indeed, the SSSG involved some unplanned jumping actions potentially leading to a post-activation phenomena (e.g., increased muscle activation) [43] that aided subsequent jumping performance during testing as previously demonstrated in rugby players [45].

While the present study offers novel insight into the efficacy of different warm-up protocols in soccer, some limitations should be acknowledged. Firstly, useful physiological measurements such as heart rate, core temperature, oxygen consumption, and blood lactate concentration were not able to be measured and therefore prevented definitive physiological reasoning from being provided to explain our findings across the different warm-up protocols. Secondly, given we examined amateur soccer players, the results cannot be simply extrapolated to players competing at higher levels due to potential differences in physical fitness and responses to warm-up activities across playing levels. Additionally, we did not examine other task constraints (e.g., touch limitation, pitch size) which could potentially affect the outcomes [46]. Finally, although the gold standard randomized, crossover design was not permissible to use in our study given players were not available for repeated assessments, we examined groups that were closely matched across various demographic and physical fitness variables following a between-groups design. Nevertheless, future studies on this topic should aim to implement randomized, crossover study designs as this approach better accounts for the inter-individual variability across players.

## CONCLUSIONS

In conclusion, both traditional and SSSG warm-up protocols improved selected physical fitness qualities, with SSSG more effective at improving CODS and CMJ performance, and TWU more effective at improving linear speed in soccer players. Soccer coaches may choose between SSSG or TWU activities according to player needs and preferences; however, the superior effects of SSSG on CODS and CMJ variables suggests it might offer greater benefits than TWU in preparing players for optimal physical output. Furthermore, coaches and practitioners could supplement the SSSG warm-up with short linear sprints to ensure linear speed is also optimized. Future studies are encouraged stemming from our work to assess whether technical abilities and tactical behaviors can be preferentially improved with specific warm-up approaches in soccer.

## References

[1] McGowan CJ, Pyne DB, Thompson KG, Rattray B. Warm-up strategies for sport and exercise: mechanisms and applications. Sports Med. 2015; 45(11):1523–46. 26400696 10.1007/s40279-015-0376-x

[2] Bishop D. Warm up I: potential mechanisms and the effects of passive warm up on exercise performance. Sports Med. 2003; 33(6):439–54. 12744717 10.2165/00007256-200333060-00005

[3] Bishop D. Warm up II: performance changes following active warm up and how to structure the warm up. Sports Med. 2003; 33(7):483–98. 12762825 10.2165/00007256-200333070-00002

[4] Hodgson M, Docherty D, Robbins D. Post-activation potentiation: underlying physiology and implications for motor performance. Sports Med. 2005; 35(7):585–95. 16026172 10.2165/00007256-200535070-00004

[5] Pardos-Mainer E., Casajús JA, Gonzalo-Skok O. Adolescent female soccer players’ soccer-specific warm-up effects on performance and inter-limb asymmetries. Biol Sport. 2019; 36(3):199–207. 31624413 10.5114/biolsport.2019.85453 PMC6786331

[6] Grandstrand SL, Pfeiffer RP, Sabick MB, DeBeliso M, Shea KG. The effects of a commercially available warm-up program on landing mechanics in female youth soccer players. J Strength Cond Res. 2006; 20(2):331–335. 16686560 10.1519/R-17585.1

[7] Fletcher IM, Monte-Colombo MM. An investigation into the effects of different warm-up modalities on specific motor skills related to soccer performance. J Strength Cond Res. 2010; 24(8):2096–101. 20634747 10.1519/JSC.0b013e3181e312db

[8] Bizzini M, Impellizzeri FM, Dvorak J, Bortolan L, Schena F, Modena R, et al. Physiological and performance responses to the “FIFA 11+” (part 1): is it an appropriate warm-up? J Sports Sci. 2013; 31(13):1481–90. 23855725 10.1080/02640414.2013.802922

[9] Zois J, Bishop DJ, Ball K, Aughey RJ. High-intensity warm-ups elicit superior performance to a current soccer warm-up routine. J Sci Med Sport. 2011; 14(6):522–8. 21907619 10.1016/j.jsams.2011.03.012

[10] Arnason A, Sigurdsson SB, Gudmundsson A, Holme I, Engebretsen L, Bahr R. Physical fitness, injuries, and team performance in soccer. Med Sci Sports Exerc. 2004; 36(2):278–85. 14767251 10.1249/01.MSS.0000113478.92945.CA

[11] Stølen T, Chamari K, Castagna C, Wisløff U. Physiology of soccer: an update. Sports Med. 2005; 35(6):501–36. 15974635 10.2165/00007256-200535060-00004

[12] Andrade DC, Henriquez-Olguín C, Beltrán AR, Ramírez MA, Labarca C, Cornejo M, et al. Effects of general, specific and combined warm-up on explosive muscular performance. Biol Sport. 2015; 32(2):123–8. 26060335 10.5604/20831862.1140426 PMC4447757

[13] Moran J, Blagrove RC, Drury B, Fernandes JFT, Paxton K, Chaabene H, et al. Effects of small-sided games vs. conventional endurance training on endurance performance in male youth soccer players: a meta-analytical comparison. Sports Med. 2019; 49(5):731–42. 30868441 10.1007/s40279-019-01086-w

[14] Jeffreys I. The use of small-sided games in the metabolic training of high school soccer players. Strength Cond J. 2004; 26(5):77–78.

[15] Clemente FM, Couceiro MS, Martins FML, Mendes R. The usefulness of small-sided games on soccer training. J Phys Edu Sport. 2012; 12(1):93–102.

[16] Clemente FM, Afonso J, Castillo D, Arcos AL, Silva AF, Sarmento H. The effects of small-sided soccer games on tactical behavior and collective dynamics: A systematic review. Chaos, Solitons & Fractals. 2020; 134:109710.

[17] Zois J, Bishop D, Aughey R. High-intensity warm-ups: effects during subsequent intermittent exercise. Int J Sports Physiol Perform. 2015; 10(4):498–503. 25393323 10.1123/ijspp.2014-0338

[18] Mallo J, Navarro E. Physical load imposed on soccer players during small-sided training games. J Sports Med Phys Fitness. 2008; 48(2):166–71. 18427410

[19] Stojanović E, Stojiljković N, Stanković R, Scanlan AT, Dalbo VJ, Milanović Z. Game format alters the physiological and activity demands encountered during small-sided football games in recreational players. J Exerc Sci Fit. 2021; 19(1):40–6. 32922462 10.1016/j.jesf.2020.05.001 PMC7475126

[20] Yanci J, Iturri J, Castillo D, Pardeiro M, Nakamura FY. Influence of warm-up duration on perceived exertion and subsequent physical performance of soccer players. Biol Sport. 2019; 36(2):125–131. 31223189 10.5114/biolsport.2019.81114 PMC6561232

[21] van den Tillaar R, von Heimburg E. Comparison of two types of warm-up upon repeated-sprint performance in experienced soccer players. J Strength Cond Res. 2016; 30(8):2258–65. 26808861 10.1519/JSC.0000000000001331

[22] van den Tillaar R, Lerberg E, von Heimburg E. Comparison of three types of warm-up upon sprint ability in experienced soccer players. J Sport Health Sci. 2019; 8(6):574–8. 31720070 10.1016/j.jshs.2016.05.006 PMC6835031

[23] Dawson B, Goodman C, Lawrence S, Preen D, Polglaze T, Fitzsimons M, et al. Muscle phosphocreatine repletion following single and repeated short sprint efforts. Scand J Med Sci Sports. 1997; 7(4):206–13. 9241025 10.1111/j.1600-0838.1997.tb00141.x

[24] Harris RC, Edwards RH, Hultman E, Nordesjö LO, Nylind B, Sahlin K. The time course of phosphorylcreatine resynthesis during recovery of the quadriceps muscle in man. Pflugers Arch. 1976; 367(2):137–42. 1034909 10.1007/BF00585149

[25] Bujalance-Moreno P, Latorre-Román P, García-Pinillos F. A systematic review on small-sided games in football players: Acute and chronic adaptations. J Sports Sci. 2019; 37(8):921–49. 30373471 10.1080/02640414.2018.1535821

[26] Bujalance-Moreno P, Latorre-Román PA, Ramírez-Campillo R, Martínez-Amat A, García-Pinillos F. The inclusion of wildcard players during small-sided games causes alterations on players’ workload. Isokinet Exerc Sci. 2021; 29:101–10.

[27] Pauole K, Madole K, Garhammer J, Lacourse M, Rozenek R. Reliability and validity of the T-test as a measure of agility, leg power, and leg speed in college-aged men and women. J Strength Cond Res. 2000; 14(4):443–50.

[28] Wee JF, Lum D, Lee M, Roman Q, Ee I, Suppiah HT. Validity and reliability of portable gym devices and an iPhone app to measure vertical jump performance. Sport Perform Sci Reports. 2018; 44(v2):1–5.

[29] Cohen J. Statistical Power Analysis for the Behavioral Sciences. Second ed. Hillsdale: Lawrence Erlbaum Associates; 1988.

[30] Hopkins WG, Marshall SW, Batterham AM, Hanin J. Progressive statistics for studies in sports medicine and exercise science. Med Sci Sports Exerc. 2009; 41(1):3–13. 19092709 10.1249/MSS.0b013e31818cb278

[31] Gray SR, Soderlund K, Watson M, Ferguson RA. Skeletal muscle ATP turnover and single fibre ATP and PCr content during intense exercise at different muscle temperatures in humans. Pflugers Arch. 2011; 462(6):885–93. 21947579 10.1007/s00424-011-1032-4

[32] Sale DG. Postactivation potentiation: role in human performance. Exerc Sport Sci Rev. 2002; 30(3):138–43. 12150573 10.1097/00003677-200207000-00008

[33] Pearce AJ, Rowe GS, Whyte DG. Neural conduction and excitability following a simple warm up. J Sci Med Sport. 2012; 15(2):164–8. 22018523 10.1016/j.jsams.2011.09.001

[34] Bergh U, Ekblom B. Influence of muscle temperature on maximal muscle strength and power output in human skeletal muscles. Acta Physiol Scand. 1979; 107(1):33–7. 525366 10.1111/j.1748-1716.1979.tb06439.x

[35] Sargeant AJ. Effect of muscle temperature on leg extension force and short-term power output in humans. Eur J Appl Physiol Occup Physiol. 1987; 56(6):693–8. 3678224 10.1007/BF00424812

[36] Racinais S, Oksa J. Temperature and neuromuscular function. Scand J Med Sci Sports. 2010; 20 Suppl 3:1–18. 10.1111/j.1600-0838.2010.01204.x 21029186

[37] Fisher M, Paolone V, Rosene J, Drury D, Van Dyke A, Moroney D. The effect of submaximal exercise on recovery hemodynamics and thermoregulation in men and women. Res Q Exerc Sport. 1999; 70(4):361–8. 10797894 10.1080/02701367.1999.10608056

[38] Poole DC, Jones AM. Oxygen uptake kinetics. Compr Physiol. 2012; 2(2):933–96. 23798293 10.1002/cphy.c100072

[39] Sander A, Keiner M, Schlumberger A, Wirth K, Schmidtbleicher D. Effects of functional exercises in the warm-up on sprint performances. J Strength Cond Res. 2013; 27(4):995–1001. 22692105 10.1519/JSC.0b013e318260ec5e

[40] Skof B, Strojnik V. The effect of two warm-up protocols on some biomechanical parameters of the neuromuscular system of middle distance runners. J Strength Cond Res. 2007; 21(2):394–9. 17530940 10.1519/R-18055.1

[41] Hammami A, Gabbett TJ, Slimani M, Bouhlel E. Does small-sided games training improve physical fitness and team-sport-specific skills? A systematic review and meta-analysis. J Sports Med Phys Fitness. 2018; 58(10):1446–55. 29072025 10.23736/S0022-4707.17.07420-5

[42] Dello Iacono A, Vigotsky AD, Laver L, Halperin I. Beneficial effects of small-sided games as a conclusive part of warm-up routines in young elite handball players. J Strength Cond Res. 2021; 35(6):1724–1731. 30741868 10.1519/JSC.0000000000002983

[43] Saez Saez de Villarreal E, González-Badillo JJ, Izquierdo M. Optimal warm-up stimuli of muscle activation to enhance short and long-term acute jumping performance. Eur J Appl Physiol. 2007; 100(4):393–401. 17394010 10.1007/s00421-007-0440-9

[44] Cilli M, Gelen E, Yildiz S, Saglam T, Camur M. Acute effects of a resisted dynamic warm-up protocol on jumping performance. Biol Sport. 2014; 31(4):277–82. 25435670 10.5604/20831862.1120935 PMC4203844

[45] Tobin DP, Delahunt E. The acute effect of a plyometric stimulus on jump performance in professional rugby players. J Strength Cond Res. 2014; 28(2):367–72. 23689338 10.1519/JSC.0b013e318299a214

[46] Younesi S, Rabbani A, Clemente FM, Sarmento H, Figueiredo AJ. Session-to-session variations in external load measures during small-sided games in professional soccer players. Biol Sport. 2021; 38(2):185–193. 34079163 10.5114/biolsport.2020.98449 PMC8139343

